# Long-chain flavodoxin FldX1 improves *Paraburkholderia xenovorans* LB400 tolerance to oxidative stress caused by paraquat and H_2_O_2_

**DOI:** 10.1371/journal.pone.0221881

**Published:** 2019-08-30

**Authors:** Laura Rodríguez-Castro, Valentina Méndez, Roberto E. Durán, Michael Seeger

**Affiliations:** Laboratorio de Microbiología Molecular y Biotecnología Ambiental, Departamento de Química & Centro de Biotecnología, Universidad Técnica Federico Santa María, Valparaíso, Chile; Universite Paris-Sud, FRANCE

## Abstract

Flavodoxins are small electron transfer proteins containing flavin mononucleotide (FMN) as a prosthetic group, which play an important role during oxidative stress or iron limitation. The aims of this study were the identification and characterization of flavodoxins in the model aromatic-degrader *Paraburkholderia xenovorans* LB400 and the analyses of their protective effects during oxidative stress induced by paraquat and H_2_O_2_. Two genes (BxeA0278 and BxeB0391) encoding flavodoxins (hereafter referred to as *fldX* for ***fl***avo***d***oxin from *P*. ***x****enovorans*), were identified at the LB400 major and minor chromosome. Genomic context of the flavodoxin-encoding genes showed genes encoding membrane proteins, transporters, and proteins involved in redox processes and biosynthesis of macromolecules. A secondary structure prediction of both LB400 flavodoxins showed the characteristic flavodoxin structure of five ß-sheets intercalated with five α-helices. FldX1 contains a loop intercalated in the fifth β-strand, which indicates that it belongs to the long-chain flavodoxins, whereas FldX2 is a short-chain flavodoxin. A phylogenetic analysis of 73 flavodoxins from 43 bacterial genera revealed eight clusters (I-VIII), while FldX1 and FldX2 grouped separately within a long-chain and a short-chain flavodoxin clades. FldX1 and FldX2 were overexpressed in *P*. *xenovorans*. Interestingly, the strain overexpressing the long-chain flavodoxin FldX1 (p2-fldX1) showed a faster growth in glucose than the control strain. The recombinant strain overexpressing the long-chain flavodoxin FldX1 (p2-*fldx1*) exposed to paraquat (20 mM) possessed lower susceptibility to growth inhibition on plates and higher survival in liquid medium than the control strain. The strains overexpressing the flavodoxins FldX1 and FldX2 showed higher survival during exposure to 1 mM paraquat (>95%) than the control strain (68%). Compared to the control strain, strains overexpressing FldX1 and FldX2 showed lower lipid peroxidation (>20%) after exposure to 1 mM paraquat and a lower protein carbonylation (~30%) after exposure to 1 mM H_2_O_2_ was observed. During exposure to paraquat, strain p2-*fldx1* downregulated the *katG4*, *hpf*, *trxB1* and *ohr* genes (> 2-fold), whereas strain p2-*fldx2* upregulated the *oxyR* and *ahpC1* genes (> 2-fold). In conclusion, the flavodoxins FldX1 and FldX2 of *P*. *xenovorans* LB400 conferred protection to cells exposed to the oxidizing agents paraquat and H_2_O_2_.

## Introduction

Microorganisms that inhabit aerobic environments are exposed to the harmful effects of reactive oxygen species (ROS), such as hydrogen peroxide (H_2_O_2_), superoxide radical (O_2_^-^) and hydroxyl radical (OH˙), which are generated by the reduction of molecular oxygen [1,2]. ROS accumulation can be potentiated by environmental factors and chemical agents, such as aromatic compounds, heavy metals and chaotropic substances [3]. The initial step in the aerobic metabolism of aromatic compounds is often the addition of molecular oxygen by oxygenases to the aromatic ring [4]. However, these enzymes may produce ROS by uncoupling of their catalytic mechanism [5,6]. Aerobic organisms possess protective mechanisms against ROS that include antioxidant and repair systems, replacement of ROS-sensitive targets by resistant isoproteins and regulation of membrane permeability. When ROS levels exceed the defense mechanisms, cells enter in a status called oxidative stress [7]. ROS may interact with macromolecules and cause protein carbonylation, lipid peroxidation and DNA mutation [1].

Flavodoxins are small electronic transfer flavoproteins, highly isofuntional with ferredoxins, expressed under oxidative stress and iron-limitation [8,9]. Ferredoxins are present in the three domains of life, whereas flavodoxins are restricted to bacteria and specific algae [7,10]. Flavodoxins and ferredoxins generally can substitute for each other, however, different enzymes preferentially use different ferredoxins/flavodoxins [11]. Ferredoxins transfer only one electron at a time, with a reduction potential E_0_′ close to −420 mV (range −340 to −500 mV). Flavodoxins also can transfer one electron with reduction potentials: E_0_′ of the quinone (Q)/semiquinone couple (SQ) is at least 200 mV more positive than E_0_′ of the SQ/quinol (HQ) couple that is −400 mV or more negative [11]. Ferredoxins possess iron-sulfur clusters (either one [2Fe-2S]-cluster or one to more [4Fe-4S]-clusters) that are sensitive to ROS, while flavodoxins have a flavin mononucleotide as a prosthetic group that is resistant to ROS. Induction of the electron shuttle flavodoxin is a common feature of the antioxidant response for cell redox balance. Flavodoxins possess a distinctive secondary structure, consisting of five ß-sheet intercalated with five α-helices [8]. Based on their sequence alignments and structures, flavodoxins are classified into short-chain and long-chain flavodoxins [3,7]. The main difference between both types of flavodoxins is a loop of approximately 20 amino acids that divide the fifth ß-sheet in long-chain flavodoxins, which is involved in the recognition of partner redox proteins [8,12].

Flavodoxins were discovered in cyanobacteria and characterized by their role in photosynthesis. Nevertheless, these proteins are involved in several processes, including nitrate reduction in *Azotobacter vinelandii* and *Klebsiella pneumoniae*, electron carrier between photosystem I and the ferredoxin-NADP(H) reductase under iron deficit in cyanobacteria *Anabaena* and *Synechocystis*, methionine biosynthesis in *Escherichia coli*, pyruvate oxidation in *Helicobacter pylori*, biotin synthesis in *Bacillus subtilis*, the reduction of oxidized metal prosthetic groups and antioxidant response in *E*. *coli* and *P*. *aeruginosa* [1,3,13]. The overexpression of the long-chain flavodoxin IsiB from the cyanobacterium *Anabaena* sp. PCC7119 in tobacco plants plastids enhances the detoxification of 2,4-dinitrotoluene and confers resistance to the redox cycling herbicide paraquat, and other stress conditions, such as extreme temperatures and high irradiation [14].

Betaproteobacteria of the *Burkholderiales* order possess a high metabolic versatility for the degradation of aromatic compounds [15,16], which are widely distributed in the environment and may be released by plants or industrial activities [17,18]. *Paraburkholderia xenovorans* LB400 is a model bacterium to study the degradation of polychlorobiphenyls (PCBs) and other aromatic compounds. *P*. *xenovorans* LB400 was isolated from a PCB-contaminated landfill in New York, United States. Strain LB400 possesses a 9.73 Mpb genome, distributed in the major chromosome (C1; 4.90 Mbp), the minor chromosome (C2; 3.36 Mbp) and the megaplasmid (MP; 1.47 Mbp) [19]. Strain LB400 genome contains several genes encoding oxygenases and hydroxylases, which are key enzymes for the degradation of aromatic compounds. Genomic analysis revealed the presence of eleven central aromatic pathways and more than twenty peripheral reactions in strain LB400 [19]. The exposure of *P*. *xenovorans* LB400 to (chloro)biphenyls (i.e., biphenyl, 4-chlorobiphenyl) and chlorobenzoates (i.e., 2-chlorobenzoate, 4-chlorobenzoate) increases the expression of molecular chaperones (DnaK, GroEL, HtpG), alkyl hydroperoxide reductase (AhpC), membrane proteins and enzymes involved in energy production, which indicates a stress response [20,21]. During *p*-cymene degradation, an increase of the molecular chaperones DnaK, GroEL and ClpB, the organic hydroperoxide resistance protein (Ohr) and AhpC was observed [22].

The aims of this study were the identification and characterization of flavodoxins of *P*. *xenovorans* LB400 and the analyses of their potential protective effect during oxidative stress induced by paraquat and H_2_O_2_. The effect of these oxidizing agents on the susceptibility and survival of *P*. *xenovorans* LB400 was studied, along with macromolecules damage and the expression of antioxidant genes.

## Material and methods

### Chemicals

Hydrogen peroxide was purchased from Merck (purity 30%; Darmstadt, Germany) and the herbicide methyl viologen dichloride hydrate (paraquat) was obtained from Sigma-Aldrich (purity 98%; St. Louis, MO, United States).

### Bioinformatic analyses

To identify genes encoding flavodoxins in the genome of *P*. *xenovorans* LB400, a DELTA-BLAST (Domain enhanced lookup time accelerated—Basic Local Alignment Search Tool) was performed [23]. The amino acid sequences of *Pseudomonas aeruginosa* PA14 (Accession No. A0A0H2ZDT7), *Escherichia coli* K12 substr. MG1655 (Accession No. P61949) and *Anabaena* sp. PCC 7119 (Accession No. P0A3E0) were used as references. The amino acid sequences of the LB400 flavodoxins was compared to the long-chain flavodoxins of *P*. *aeruginosa* PA14 (Accession No. A0A0H2ZDT7) and FldA of *E*. *coli* K12 substr. MG1655 (Accession No. P61949), and the short-chain flavodoxins of *Clostridium beijerinckii* MP (Accession No. P00322), and *Paraburkholderia phymatum* TM815 (Accession No. B2JVA6) using the pairwise sequence alignment tool EMBOSS Needle (https://www.ebi.ac.uk/Tools/psa/emboss_needle/). The PROSITE tool of Expasy [24] was used to search for amino acid conserved domains. The secondary structure was predicted using the Phyre2 [25] and PROMALS3D [26] tools and compared with the long-chain flavodoxins of *P*. *aeruginosa* PA14 (Accession No. A0A0H2ZDT7), *E*. *coli* K12 substr. MG1655 (Accession No. P61949) and *Anabaena* sp. PCC 7119 (Accession No. P0A3E0), and the short-chain flavodoxins of *C*. *beijerinckii* MP (Accession No. P00322), *Desulfovibrio desulfuricans* ATCC 29577 (Accession No. P26492) and *Bacillus subtilis* 168 (Accession No. O34737). The identity between LB400 flavodoxins was determined by the pairwise sequence alignment tool EMBOSS Needle (https://www.ebi.ac.uk/Tools/psa/emboss_needle/). The genomic context was analyzed through the *Burkholderia* database (http://www.burkholderia.com/).

### Bacterial strains and culture conditions

*P*. *xenovorans* LB400 was grown at 30°C in Luria-Bertani (LB) modified medium (5 g L^-1^ tryptone, 2.5 g L^-1^ yeast extract and 2.5 g L^-1^ NaCl) or in M9 minimal medium with 5 mM glucose as sole carbon and energy source. *E*. *coli* strains were growth at 37°C in LB medium. Growth was determined by measuring turbidity at 600 nm and/or by counting colony-forming units (CFU).

### Phylogenetic analysis of *P*. *xenovorans* LB400 flavodoxins

Thirty-nine flavodoxin amino acid sequences with experimental evidence were retrieved from UniProtKB Swiss-Prot/TrEMBL database [27]. Identification of FldX1 and FldX2 homologs was performed using the DELTA-BLAST protein homology search using both proteins as queries. A total of 73 flavodoxins amino acid sequences, including FldX1 and FldX2, were aligned using MAFFT version 7.407 [28]. The alignment was manually trimmed using Aliview version 1.24 [29]. The best evolutionary model was identified using the program PartitionFinder version 2.1.1 [30]. A distribution of probable trees was obtained, by Bayesian Inference as implemented in MrBayes 3.2.6 [31]. Three separate runs of 1 million generations were executed (two chains each run; sampling every 1,000 generations). Visualization and editing of phylogenetic trees were performed using the FigTree v. 1.4.2 software (http://tree.bio.ed.ac.uk/software/figtree/). The bootstrap values (percentage) above 50% were shown for each branch point.

### Construction of recombinant strains

Genes encoding flavodoxins (BxeA0278 and BxeB0391) of *P*. *xenovorans* LB400 were amplified by PCR using specific primers (Table 1). The PCRs products and the plasmid pBBR1MCS-2 (hereafter p2) [32] were digested with the *BamH*I and *Hind*III (New England Biolabs; Ipswich, MA, USA) restriction enzymes. The DNA fragments were cloned using T4 DNA ligase (Promega; Madison, WI, USA) into the vector p2, which possesses a kanamycin resistance cassette. The ligation reactions were transformed into *E*. *coli* JM109 and cells were cultured overnight with kanamycin 25 μg mL^-1^ as selective marker. Positive clones were checked by enzymatic digestion with *BamH*I and *Hind*III and PCR (Table 1), using the same primers used for cloning. Gene sequences were checked by Sanger sequencing. Clones for FldX1 and for FldX2 were selected. To obtain the *P*. *xenovorans* LB400 strains overexpressing the flavodoxins, the recombinant plasmids were transformed into *E*. *coli* S17λpir and transferred to *P*. *xenovorans* LB400 by biparental mating. Recombinant strains (hereafter p2-*fldX1* and p2-*fldX2*) were checked by PCR using the M13 primers. The overexpression of the FldX1 and FldX2 proteins was confirmed by SDS-PAGE 15% w v^-1^. To construct the control strain (hereafter WT-p2), the vector p2 without insert was transformed into *E*. *coli* S17λpir and transferred to *P*. *xenovorans* LB400 by biparental mating. The recombinant strain was checked by PCR using M13 primers. The expression level of genes encoding flavodoxins in recombinant strains was determined by qRT-PCR (Table 1).

**Table 1:** Oligonucleotides used as primers in this study.

Target	Primer	Sequence 5´-3´	Reference
*fldX1*	FldA_FW	CGCGGATCCATGTCGAAGATCGTCATCGTT	This study
	FldA_RV	TATAAGCTTTCAGCGGGCCGCGGCGAT	
*fldX2*	FldB_FW	CGCGGATCCATGTCGGATCAATACAAA	This study
	FldB_RV	CCAAGCTTGGCTATGCCGCACATTCCATTG	
*fldX1*	FldX1_qPCR_FW	CAGTATTTCGTCACGCTGGC	This study
	FldX1_qPCR_RV	GTAAAGCCGCCCACGTAGTT	
*fldX2*	FldX2_qPCR_FW	CCTGTGCCTTTGATGCGTCC	This study
	FldX2_qPCR_RV	CATTCCATTGCCAGCAGCGT	
Mlti-cloning site plasmid p2	M13F (-20)	GTAAAACGACGGCCAG	[33]
	M13R (-20)	GCGGATAACAATTTCACACAGG	
*fumC*	FumA1038Fw	CGTACGAATGGAGCGTGACA	[34]
	FumA1038Rv	ATGAGTTCGGGCGATTGCTT	
*sodB1*	SodA1769Fw	GCGGCTCAAGTGTGGAATCA	[34]
	SodA1769Rv	CTGCGGTCTTGGCGA ATTCT	
*oxyR*	OxA39Fw	GAAGCGTGTTTCGTCAGCCA	[35]
	OxA39Rv	TTCGAGGACACGTTGAGCTT	
*ahpC1*	AhpA2309Fw	GCGTCGACAACGAATTCGTG	[34]
	AhpA2309Rv	TCGATCAGCTCGCCTTTCAC	
*katG4*	KatB1215Fw	TCATCGAGGAAGCGGACGAA	[34]
	KatB1215Rv	TGTCCGGATTGCGATTGAGC	
*hpf*	HpfFw	CGAGCAAAGTCGACAAAGCG	[35]
	HpfRv	TATATGCGCTGCACCAACCC	
*txrB1*	A3443TrFw	CCCATGAACGCTTCTTCCGA	[35]
	A3443TrRv	ACCACATTCACACGGCAAAG	
*txrB2*	A3962TxFw	GACCGGTGTCGATTGGTGTT	[35]
	A3962TxRv	ATAAACCCGTGCTGCTCGATT	
*gst*	GstA0624Fw	GCGACAGCGTATTCCAGGTATT	[35]
	GstA0624Rv	CTGGTGCCCCAGAATGTCTG	
*ohr*	OhrB2843Fw	CCCGGCGACAAACTTCATTG	[35]
	OhrB2843Rv	GGCGGTGTGTCGGATAAT	
*ftsZ*	FtsZFw	CGATTACGGTGCGCTGCATA	[35]
	FtsZRv	ATGCCGGAATGTCGTACGTG	
*gyrR*	GyrFw	GGGCAAGGACGAACGGTATT	[35]
	GyrRv	ACAGATAAGCCCGAGCCAAC	

### Growth assays

To study the bacteria growth of the recombinant strains, cells were grown in 5 mM glucose. Recombinant strains were growth overnight in modified LB medium at 30°C. Cells were washed in 0.9% NaCl and used as inoculum for M9 minimal medium with 5 mM glucose as sole carbon source. Recombinant strains were cultivated for 24 h at 30°C and turbidity a 600 nm was measured every 3 h.

### Susceptibility assays

Recombinant strains were grown until exponential phase and 100 μL of each culture was spread on modified LB medium agar plates. 15-μL of hydrogen peroxide (H_2_O_2_; 20 mM) or the superoxide-producing agent paraquat (20 mM) were deposited on 6 mm diffusion discs and placed on the plates. Images of the growth inhibition zones were measured using the software ImageJ after 24 h incubation at 30°C [36]. Values were calculated as the mean ± SD of results of, at least, three independent experiments.

### Survival of recombinant strains

Exponential grown cells of *P*. *xenovorans* recombinant strains were harvested and washed with 0.9% w v^-1^ NaCl. Cells were incubated in presence of H_2_O_2_ or paraquat (1 mM and 20 mM) during 1 h. Aliquots were taken to determine colony-forming units. The percentage of survival was determined as the ratio of cells exposed to oxidizing agents and untreated cells. Values were calculated as the mean ± SD of results of, at least, three independent experiments.

### Protein carbonylation assays

Exponentially-grown cells of *P*. *xenovorans* recombinant strains were exposed to 1 mM H_2_O_2_ or 1 mM paraquat during 1 h at 30°C. Bacterial cells were harvested and disrupted by sonication using five pulses of 30 s at 6 Watts with 1-min intervals. Nucleic acid-free extracts were used to precipitate proteins with 10% v v^-1^ trichloroacetic acid. 400-μL of protein extracts were incubated with 400 μL of 10 mM 2,4-dinitrophenylhydrazine (DNPH) during 10 min. 200-μL of 6 M NaOH was added to the reaction and incubated for 10 min at room temperature. Carbonyl content was determined spectrophotometrically at 450 nm using a molar absorption coefficient of 22,308 M^-1^cm^-1^ [37]. Values were calculated as the mean ± SD of results of, at least, three independent experiments.

### Determination of thiobarbituric acid reactive substances (TBARS)

Exponentially-grown cultures of *P*. *xenovorans* recombinant strains were exposed to H_2_O_2_ or paraquat (1 mM) for 1 h. Bacterial cells were harvested and disrupted by sonication. Proteins were precipitated with 10% v v^-1^ trichloroacetic acid and discarded by centrifugation. Supernatants were mixed with a saturated solution of thiobarbituric acid (TBA) in 0.1 M HCl and 10 mM butylated hydroxytoluene. Samples were incubated at 100°C for 1 h. TBA reacted with malondialdehyde and other byproducts of lipid peroxidation forming a colored product. TBARS content was determined spectrophotometrically at 535 nm using a molar absorption coefficient of 156 mM^-1^cm^-1^ [38]. Values were calculated as the mean ± SD of results of, at least, three independent experiments.

### Gene expression analysis

Differential expression of genes was analyzed by qRT-PCR. For the quantification of the expression of flavodoxins genes, *P*. *xenovorans* recombinant strains were grown until mid-exponential phase (turbidity_600nm_ ~0.4) and harvested. For the quantification of the expression of genes associated to antioxidant response, exponentially-grown cells (turbidity_600nm_ ~0.4) were exposed to 1 mM paraquat during 1 h and harvested. RNA was extracted from cells using the TRIzol reagent (Thermo Fisher Scientific; Waltham, MA, USA), following the manufacturer instructions. Residual DNA was removed employing the TURBO DNA-free Kit (Thermo Fisher Scientific; Waltham, MA, USA). RNA quality was verified with the NanoDrop One spectrometer (Thermo Fisher Scientific; Waltham, MA, USA). RNA integrity was checked in a 1% w v^-1^ agarose gel. cDNA was synthetized with the First Strand cDNA Synthesis Kit (Thermo Fisher Scientific; Waltham, MA, USA). The qRT-PCRs were carried out with the KAPA SYBR FAST qPCR Master Mix Kit (Kapa Biosystems; Boston, MA, USA), following the manufacturer instructions. The *gyrB* and *ftsZ* genes were used as reference genes. The results were analyzed using the Hellemans method [39]. Untreated cells were used as control. Negative and positive controls were included in each RT-PCR assay. At least three independent RNA samples were analyzed at each condition and three independent RT-PCR reactions for each sample were done to assess reproducibility. The primers used in this study are listed in Table 1.

### Statistical analysis

Statistical analyses were performed using one-way ANOVA and Tukey test to assess differences in mean values from each experiment. Differences are significant at p<0.05.

## Results

### Identification and characterization of flavodoxins of *P*. *xenovorans* LB400

For the identification of genes encoding flavodoxins in the genome of *P*. *xenovorans* LB400, amino acid sequences of three well-characterized flavodoxins were retrieved from the UniProt database: *P*. *aeruginosa* PA14 (Accession No. A0A0H2ZDT7), *E*. *coli* K12 substr. MG1655 (Accession No. P61949) and *Anabaena* sp. PCC 7119 (Accession No. P0A3E0). Two putative genes encoding flavodoxins were identified in the genome of *P*. *xenovorans* LB400. The BxeA0278 gene (hereafter *fldX1*) localized at the major chromosome encodes a protein of 190 amino acids. The BxeA0278 gene product showed 50, 26 and 23% identity with the FldP, FldA and IsiB flavodoxins of *P*. *aeruginosa* PA14, *E*. *coli* K12 and *Anabaena* sp. PCC7119, respectively. The BxeB0391 gene (hereafter *fldX2*), is located at the minor chromosome and encodes a protein of 175 amino acids. The BxeB0391 gene product possesses 44, 21 and 21% identity with FldP, FldA and IsiB, respectively. Genes encoding LB400 flavodoxins was compared with the long-chain flavodoxins of *P*. *aeruginosa* PA14 (Accession No. A0A0H2ZDT7) and *E*. *coli* K12 substr. MG1655 (Accession No. P61949), and the short-chain flavodoxins of *C*. *beijerinckii* MP (Accession No. P00322) and *P*. *phymatum* TM815 (Accession No. B2JVA6) (Table 2). Interestingly, flavodoxin FldX1 possess a higher identity (48.7%) with the long-chain flavodoxin of *P*. *aeruginosa* PA14, while FldX2 showed a higher identity with the short-chain flavodoxin of *P*. *phymatum* TM815. This may indicate that FldX1 is a long-chain flavodoxin and FldX2 is a short-chain flavodoxin.

**Table 2:** Comparison of flavodoxins identified in *P*. *xenovorans* LB400 genome with long-chain and short-chain flavodoxins.

Gene	Protein (aa)	Organism (gene)^a^	Amino acids	Identity (%)	Similarity (%)	Score	Accesion No.
BxeA0278 *(fldX1)*	Flavodoxin 190	*P*. *aeruginosa* PA14 (*fldP*)	184	48.7	64.2	458	A0A0H2ZDT7
		*E*. *coli* K12 substr MG1655 (*fldA*)	176	20.8	32.9	58	P61949
		*C*. *beijerinckii* MP	138	20.7	38.9	91	P00322
		*P*. *phymatum* TM815	175	10.9	18.0	64	B2JVA6
BxeB0391 *(fldX2)*	Flavodoxin 175	*P*. *phymatum* TM815	175	59.4	68.0	492	B2JVA6
		*E*. *coli* K12 substr MG1655 (*fldA*)	176	19.7	31.2	52	P61949
		*P*. *aeruginosa* PA14 (*fldP*)	184	17.7	29.6	47	A0A0H2ZDT7
		*C*. *beijerinckii* MP	138	12.7	21.8	46	P00322

^a^ Sequences of previously described flavodoxins were used to perform DELTA-BLAST.

To determine conserved domains, the primary structures of strain LB400 flavodoxins were analyzed using the PROSITE tool (Expasy). FldX1 possesses a flavodoxin domain between amino acids 4 and 179, and two FMN-binding domains (FMN-BD) between amino acids 10–14 and 93–150. The amino acid sequence of the first FMN-BD (SGYGH) domain is identical to the FldP flavodoxin of *P*. *aeruginosa* PA14. This FMN-BD domain is located in the same positions than in FldP of *P*. *aeruginosa* PA14 and FldA of *E*. *coli* K12. FldX2 possess a flavodoxin domain between amino acids 7 and 175, and two FMN-BD domains between amino acids 13–17 and 132–175.

**Fig 1:**
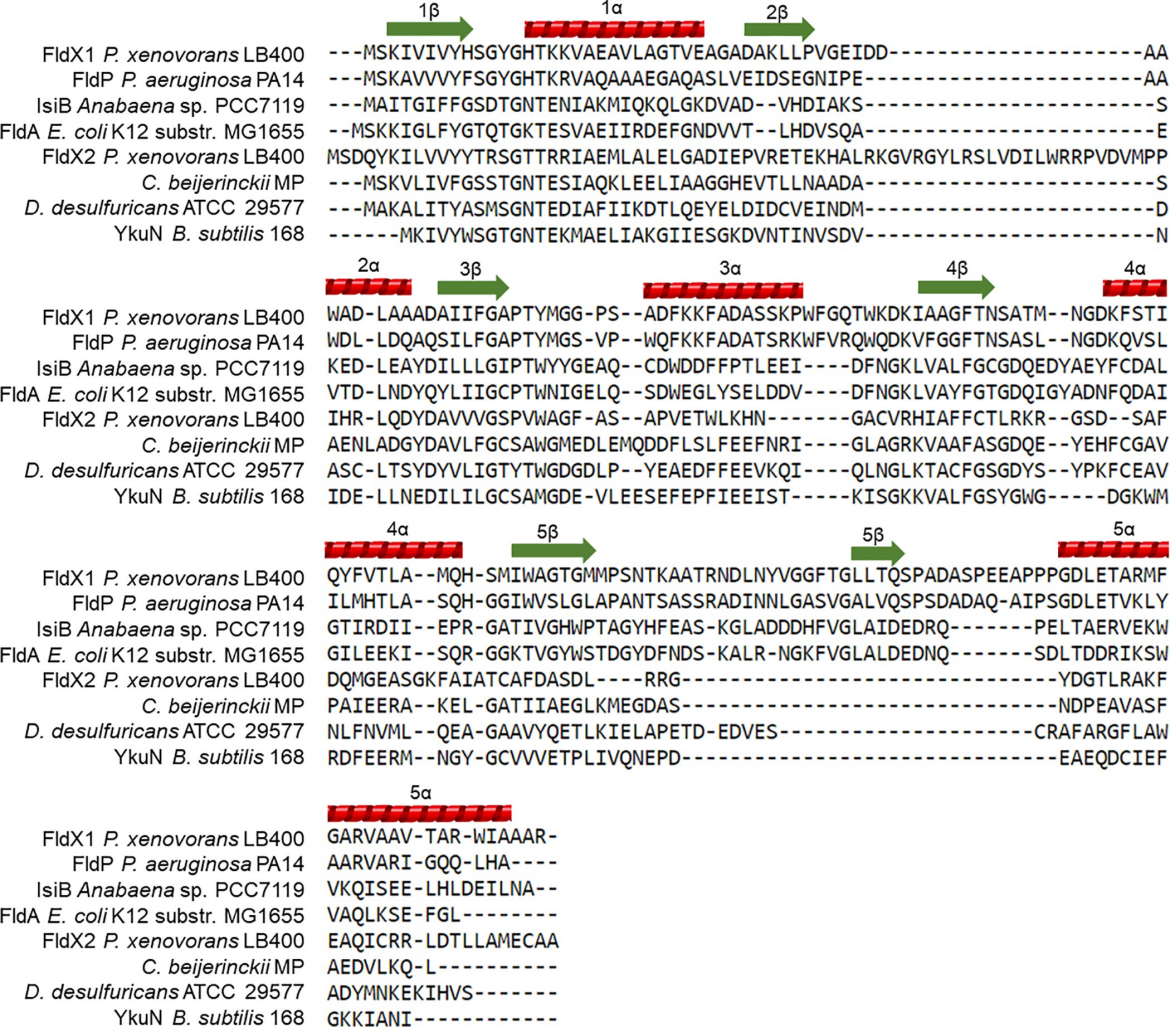
Prediction of secondary structure of LB400 flavodoxins. Green arrows indicates β-sheets and red helices indicates α-helices.

The secondary structures of both flavodoxins were predicted using the Phyre2 and PROMALS3D tools (Fig 1). FldX1 and FldX2 exhibited the characteristic secondary structure traits of flavodoxins, consisting of 5 ß-sheet intercalated with 5 α-helices. However, only FldX1 possesses an insertion loop that divides the fifth ß-sheet. This suggest that FldX1 is a long-chain flavodoxin, while FldX2 is a short-chain-flavodoxin. A pairwise sequence alignment analysis revealed 21.4% aminoacidic sequence identity between flavodoxins FldX1 and FldX2.

**Fig 2:**
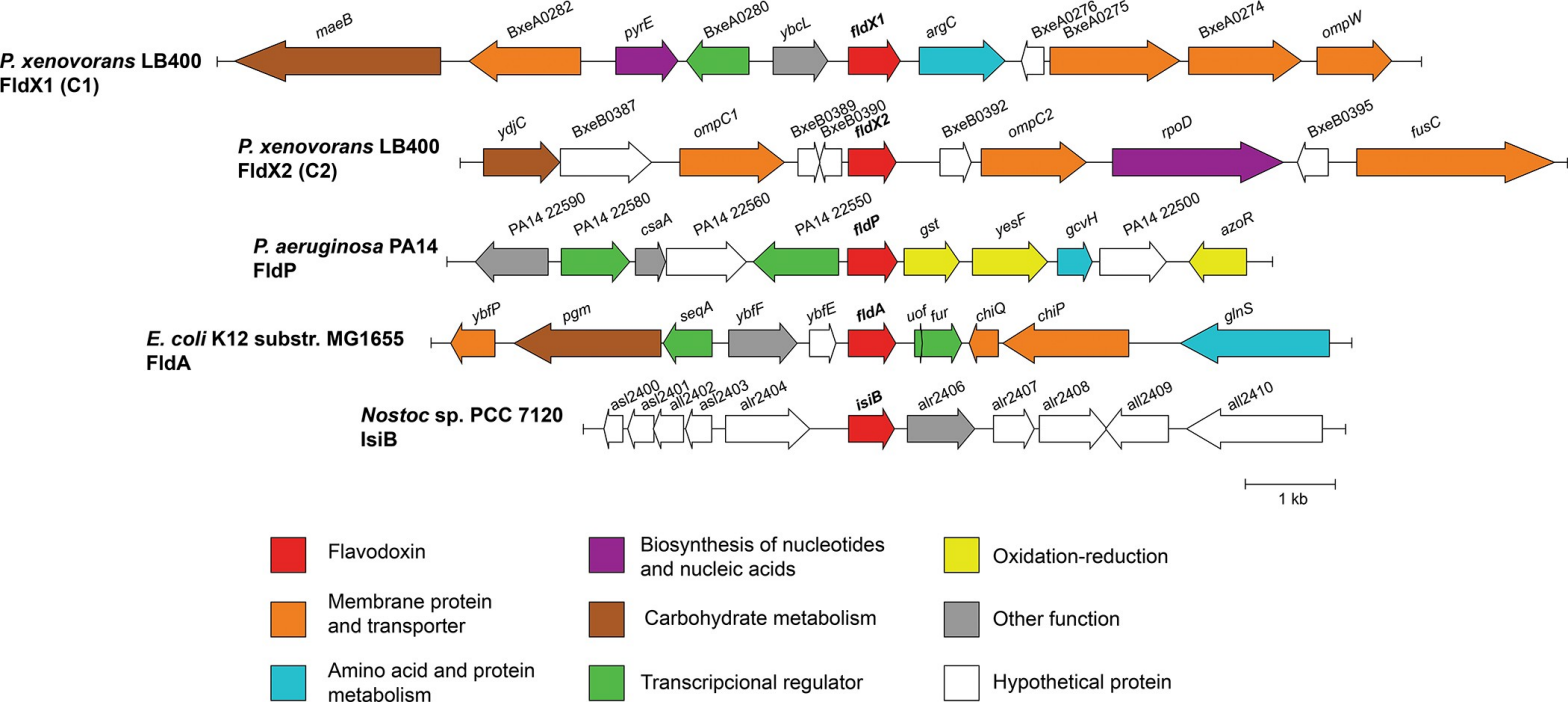
Genomic context of flavodoxins of *P*. *xenovorans* LB400. Gene encoding FldX1 is located in the major chromosome, while FldX2-encoding gene is located in the minor chromosome of strain LB400. Five genes upstream and five downstream of flavodoxin coding gene are shown. For comparison, the genomic context of other three flavodoxins with assigned biological functions is shown.

The neighborhoods of the *fldX1* and *fldX2* genes in strain LB400 genome were analyzed. Genes encoding membrane proteins, transporters, proteins involved in redox processes, biosynthesis of nucleic acids, transcriptional regulators, metabolism of amino acids, proteins and carbohydrates were identified in neighborhoods of the *fldX1* and *fldX2* genes in strain LB400 genome and of FldP, FldA and IsiB flavodoxins genes of *P*. *aeruginosa* PA14, *E*. *coli* K12 (MG1655) and *Nostoc* sp. PCC 7120 (*Anabaena*), respectively (Fig 2). Nucleic acids and proteins might be damaged by ROS in cells subjected to oxidative stress [1,2]. Therefore, the genes encoding proteins involved in the synthesis of nucleic acids and proteins including the replacement of damaged macromolecules may be useful during oxidative stress.

In the *fldX1* gene neighborhood are located genes encoding the membrane proteins OmpW family porin (*ompW*), two membrane lipoproteins (BxeA0274 and BxeA0275) and a major facilitator transporter (BxeA0282). The *ompW* gene located downstream of the *fldX1* gene encodes the OmpW porin that may be involved in paraquat efflux in strain LB400. The OmpW porin mediates paraquat efflux in *Salmonella enterica* serovar typhimurium [40]. Upstream of the *fldX1* gene is located a LuxR family transcriptional regulator gene (BxeA0280). It has been proposed that the LysR family regulator of *P*. *aeruginosa* PA14 encoded by a gene located close to the *fldP* gene is the repressor of the transcription of the FldP operon [7]. The LB400 LuxR family transcriptional regulator (BxeA0280) may regulate the expression of the *fldX1* gene, however, experimental assays are required to study the regulation. In addition, the gene *argC* (BxeA0277) encoding N-acetyl-γ-glutamyl-phosphate reductase is in the context of the *fldX1* gene. N-acetyl-γ-glutamyl-phosphate reductase ArgC is involved in the synthesis of arginine and proline from glutamate [41]. Arginine, proline, histidine, lysine, threonine and tryptophan are the main amino acids targets for oxidation by ROS [42], which may explain the presence of the *argC* gene in the neighborhood of the *fldX1*gene. In addition, in the *fldx1* gene neighborhood are located genes encoding an orotate phosphoribosyl-transferase (*pyrE*; BxeA0281) that is involved in nucleotide biosynthesis, a phospholipid-binding protein (*ybcL*; BxeA0279) and the malic enzyme (*maeB*; BxeA0283).

In the *fldX2* gene neighborhood are located two genes encoding the membrane proteins OmpC family porins (*ompC1* and *ompC2*; BxeB0288 and BxeB0392, respectively) and a gene encoding an aromatic acid exporter (*fusC*; BxeB0396). The presence of porins and aromatic exporter may explain in part the bacterial resistance to antibiotics and toxic compounds such as paraquat [40]. In addition, a chitooligosaccharide deacetylase (*ydjC*; BxeB0286) involved in carbohydrate metabolism and a gene encoding the RNA polymerase sigma 70 factor (*rpoD*; BxeB0294) was identified in the *fldX2* gene neighborhood.

The long-chain flavodoxin NifF genes of nitrogen-fixating *A*. *vinelandii* OP and *P*. *stutzeri* A1501 are clustered with genes involved in nitrogen fixation [43,44], which is consistent with the interaction of the flavodoxin with the nitrate reductase [8]. The genomic context of *P*. *xenovorans* LB400 flavodoxins genes reveals genes encoding proteins involved in the synthesis of nucleic acids and proteins and efflux of toxic compounds, which might influence bacterial fitness during oxidative stress.

### Phylogenetic analysis of FldX1 and FldX2 flavodoxins

**Fig 3:**
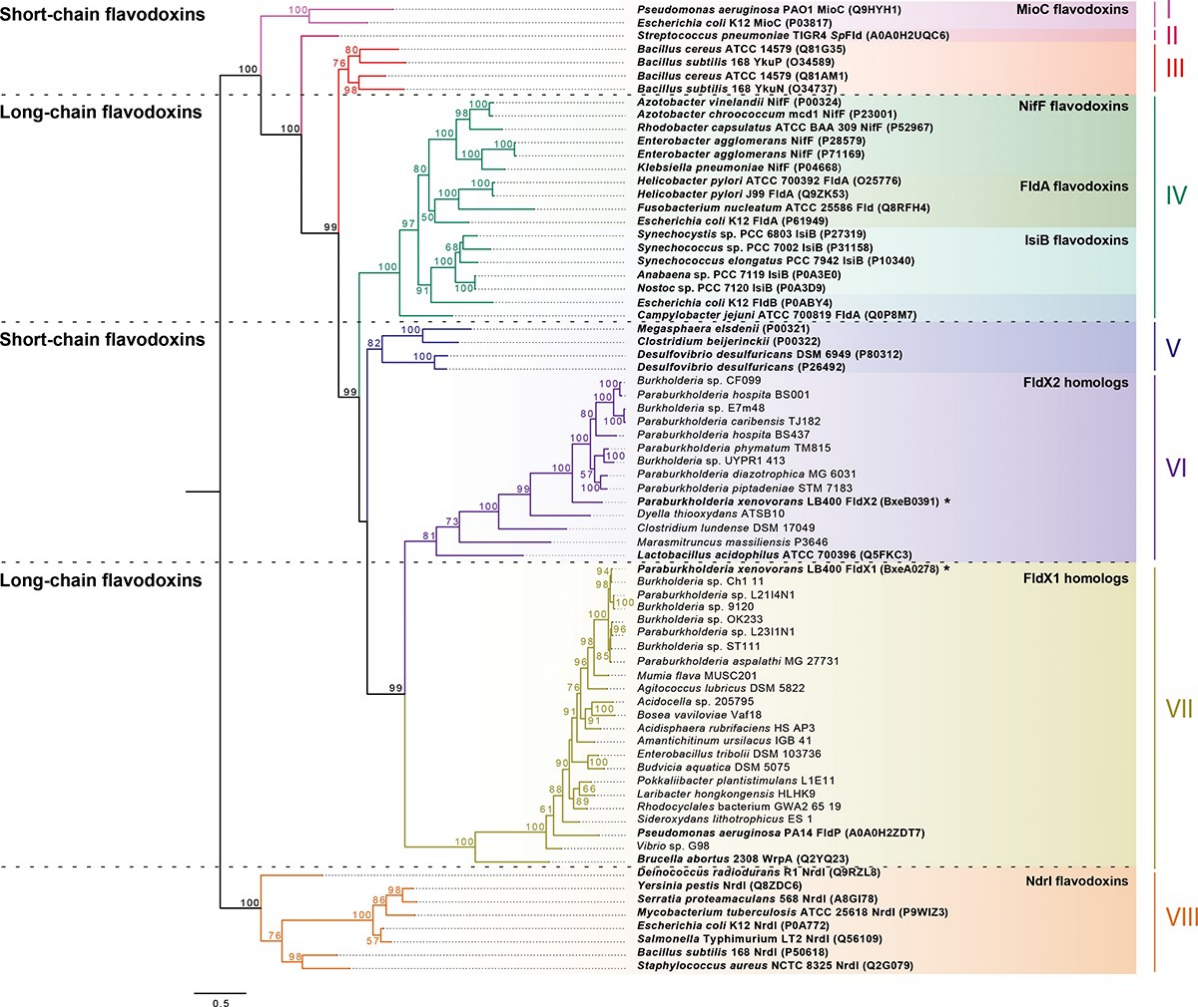
Phylogenetic analysis of *P*. *xenovorans* LB400 flavodoxins. NrdI flavodoxins rooted phylogeny showed 8 defined clusters (I-VIII). FldX1 and FldX2 were designated with asterisks (*). Bold letters denote experimentally proven flavodoxins. The bootstrap values (percentage) above 50% were shown for each branch point. FldX1 groups with FldP of *P*. *aeruginosa* PA14 and WrpA of *B*. *abortus* 2308 (cluster VII), while FldX2 groups with flavodoxin-4 of *L*. *acidophilus* ATCC 700396 positioned in the cluster VI.

To further understand the evolutionary relationships of *P*. *xenovorans* LB400 flavodoxins, the phylogeny of FldX1, FldX2 and 71 flavodoxins from 43 different bacterial genera was analyzed. From this dataset, 20 amino acid sequences correspond to FldX1 orthologs, while 12 belong to FldX2 orthologs. A total of 39 flavodoxins with experimental evidence were incorporated to evaluate FldX1 and FldX2 evolutionary position among flavodoxins with an assigned biological function. The phylogenetic tree was rooted using the NrdI flavodoxin family, involved in ribonucleotide reductase function, showing eight distinct clusters (Fig 3).

As expected, the NifF flavodoxins involved in reduction of nitrate by nitrogenase belonging to the *Azotobacter*, *Enterobacter*, *Klebsiella* and *Rhodobacter* genera; the IsiB flavodoxins participating as an electron carrier under iron-deficient conditions of the cyanobacteria *Anabaena*, *Nostoc*, *Synechococcus* and *Synechocystis*; and the FldA flavodoxins of *Escherichia*, *Campylobacter* and *Helicobacter* formed distinct clades among the first cluster of long-chain flavodoxins identified (cluster IV; Fig 3). This clustering is in accordance with the previous study of Pérez-Dorado *et al*. [13]. The cluster VII comprising long-chain flavodoxins contains mainly *Proteobacteria* flavodoxins, including the flavodoxins FldX1 of *P*. *xenovorans* LB400, FldP of *P*. *aeruginosa* PA14 and the atypical WrpA flavodoxin of *Brucella abortus* 2308 (cluster VII; Fig 3).

Five defined clusters of short-chain flavodoxins containing amino acid sequences of Gram-positive and -negative bacteria were identified (clusters I, II, III, V and VI, Fig 3). The first cluster includes the amino acid sequences of MioC flavodoxins of *E*. *coli* K12 and *P*. *aeruginosa* PAO1 (Fig 3). Two clusters containing the Gram-positive flavodoxin sequences of the *Bacillus* and *Streptococcus* genera were identified (cluster II and III, Fig 3). Cluster V contained a mixed clade of Gram-positive and -negative flavodoxins containing the amino acid sequences of *Clostridium*, *Megasphaera* and *Desulfovibrio* genera. A cluster of short-chain flavodoxins contained the FldX2 flavodoxin from strain LB400, its homologs from the *Burkholderiales* and *Clostridiales* orders and the flavodoxin-4 from *Lactobacillus acidophillus* ATCC 700396 that belong to the *Firmicutes* phylum (cluster VI; Fig 3). Finally, the NrdI flavodoxins belonging to the *Bacillus*, *Deinococcus*, *Escherichia*, *Mycobacterium*, *Salmonella*, *Serratia*, *Staphylococcus* and *Yersinia* genera grouped together in the cluster VIII.

### Growth assays

To characterize the growth of *P*. *xenovorans* recombinant strains, cells were cultured in M9 minimal medium with 5 mM glucose as sole carbon and energy source. Strain p2-*fldX1* overexpressing the long-chain flavodoxin FldX1 showed faster aerobic growth on glucose compared to control strain. The growth of strains p2-*fldX2* and control strain were similar. Strain p2-*fldx1* showed the highest growth rate (0.081 h^-1^) on glucose, while no differences were detected between strains p2-*fldx2* and control (0.051 and 0.053 h^-1^, respectively). These results suggest that overexpression of flavodoxin FldX1 is conferring an advantage during aerobic growth on glucose.

### Transcription of the *fldX* genes

The transcription of the *fldX* genes in recombinant strains was studied by qRT-PCR using specific primer sets (Table 1). Recombinant strains were cultured in M9 minimal medium with 5 mM glucose as sole carbon and energy source until mid-exponential phase (turbidity_600nm_ ~0.4). The *fldX1* gene expression was highly induced (>600-fold) in recombinant strain p2-*fldX1* compared to the control WT-p2 strain. In recombinant strain p2-*fldX2*, the *fldX2* gene expression was >4000-fold induced compared to the control WT-p2 strain. Interestingly, despite that the genes encoding both flavodoxins were cloned in the same plasmid, the overexpression of *fldX2* gene in strain p2-*fldX2* is higher (>6-fold) than the overexpression of *fldX1* gene in recombinant strain p2-*fldX1*. These results suggest a higher expression of the *fldX1* gene than the *fldX2* in WT-p2 strain grown on glucose.

### Susceptibility assays

**Fig 4:**
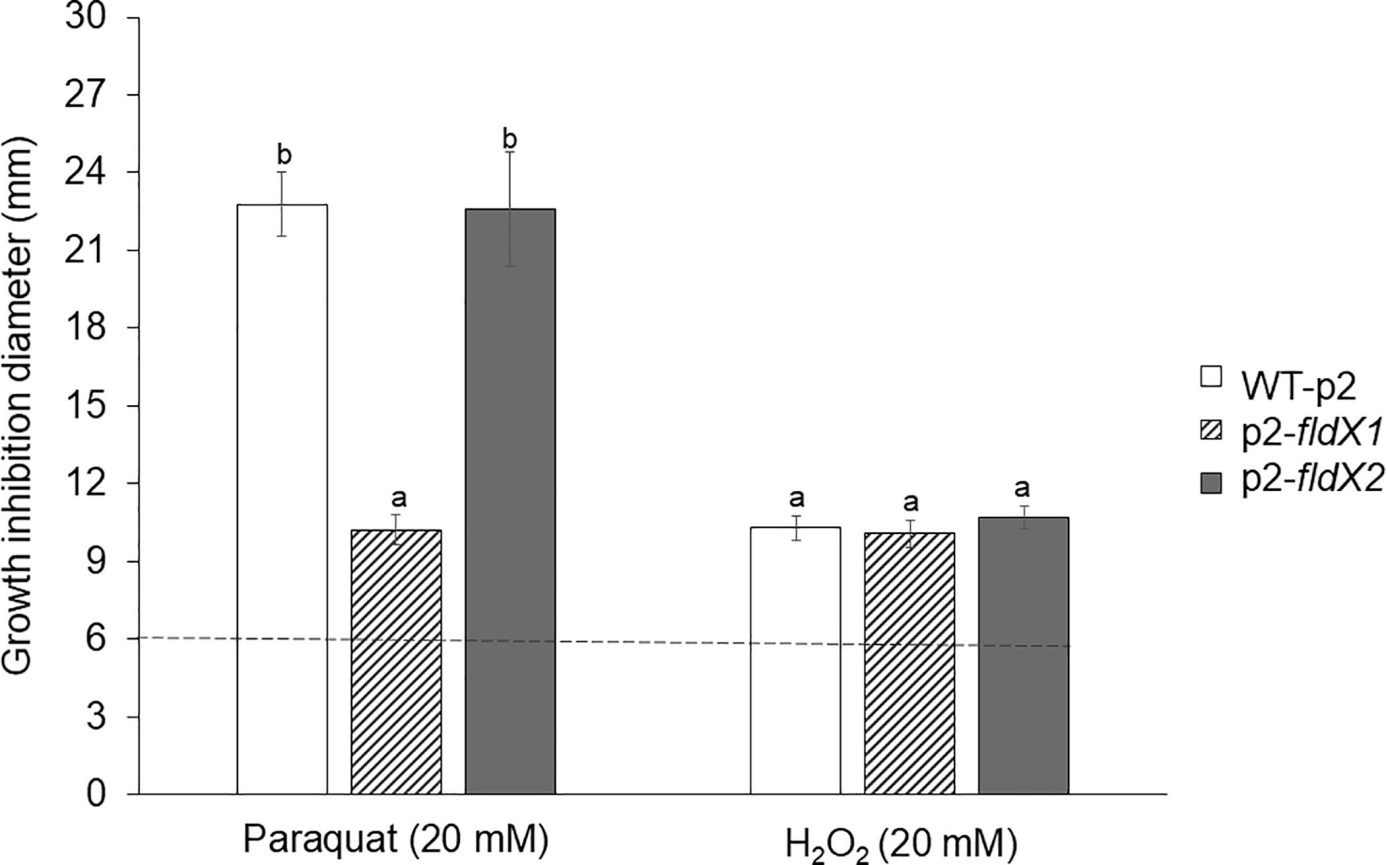
Effects of the flavodoxins on the *P*. *xenovorans* susceptibility to oxidizing agents paraquat and H_2_O_2_ on agar plates. Cells grown until exponential phase were platted, and discs containing 20 mM H_2_O_2_ or 20 mM paraquat were disposed on the plate. Growth inhibition zones were measured after 24 h incubation at 30ºC. The inhibition zone of 6 mm corresponds to the disc diameter without oxidizing agent (control). Significant differences were determined with a Tukey Test (p<0.05) as indicated. Each value is an average ± SD of at least three independent experiments.

The susceptibility of the *P*. *xenovorans* strains overexpressing *fldX1* and *fldX2* genes to oxidizing agents was assessed. Recombinant *P*. *xenovorans* cells on agar plates were exposed to the superoxide-producing redox-cycling agent paraquat or H_2_O_2_ (20 mM) (Fig 4). Interestingly, after incubation with 20 mM paraquat, the recombinant strain overexpressing the *fldX1* gene, showed a significant smaller inhibition zone (10.2 mm) compared to the control strain (22.8 mm). In contrast, after incubation with paraquat the strain overexpressing the *fldX2* gene did not show significant differences with the control strain (22.6 mm). After incubation with 20 mM H_2_O_2_, the strains overexpressing the *fldX1* (10.1 mm) and *fldX2* genes (10.7 mm) did not show significant differences with the control strain (10.3 mm).

### Survival to oxidizing agents

**Fig 5:**
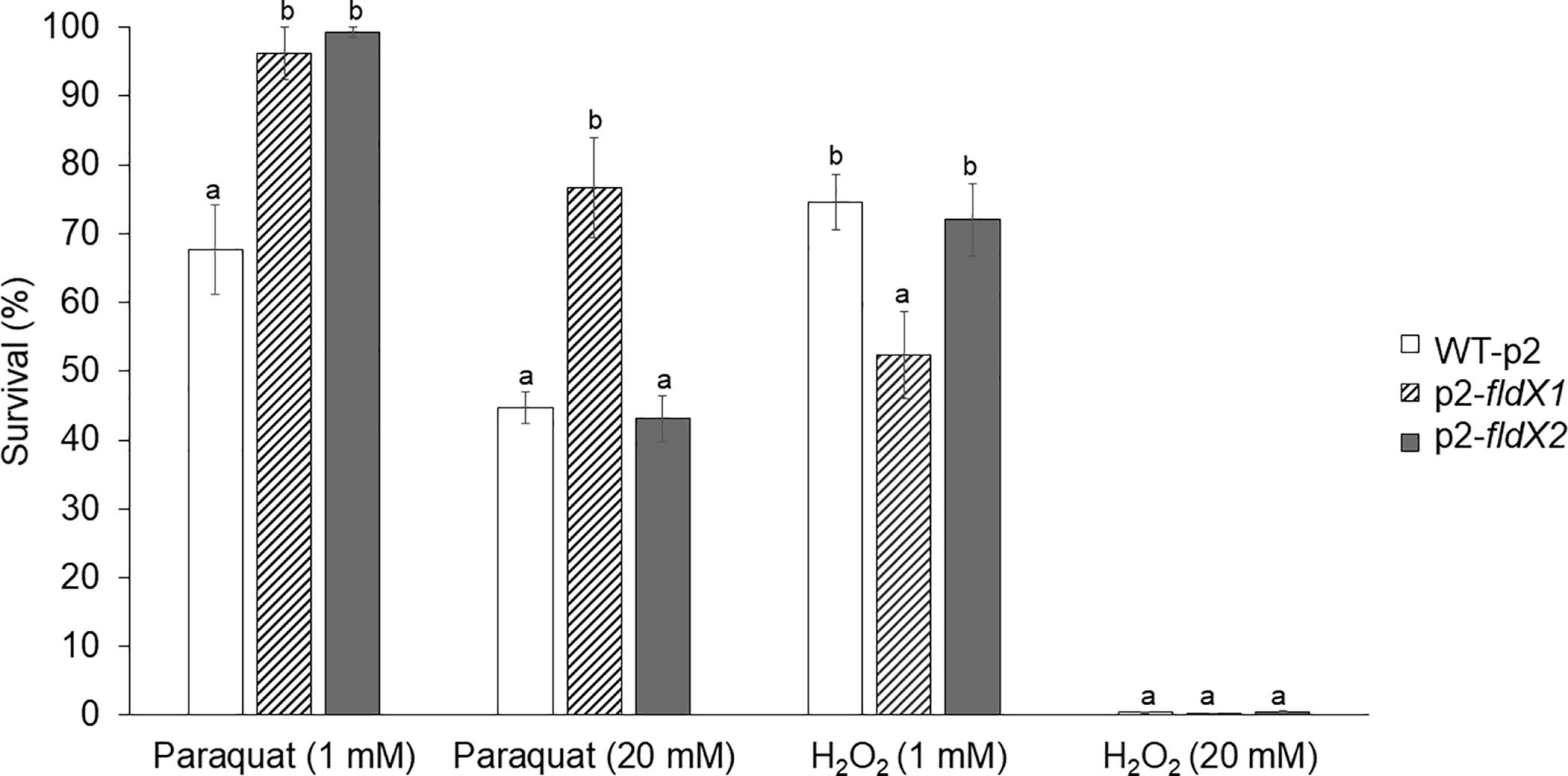
Effects of the flavodoxins on *P*. *xenovorans* survival after exposure to oxidizing agents paraquat and H_2_O_2_ in liquid medium. Exponentially grown cells were exposed to paraquat or H_2_O_2_ (1 and 20 mM) for 1 h. Aliquots were taken to determine colony-forming units (CFU). The percentage of survival was determined as the ratio of cells exposed to oxidizing agents and untreated cells. Significant differences were determined with a Tukey Test (p<0.05) as indicated. Each CFU value is an average ± SD of at least three independent experiments.

The protective effects of FldX1 and FldX2 on *P*. *xenovorans* during exposure to oxidizing agents was studied by survival assays in liquid medium. Recombinant *P*. *xenovorans* cells grown until exponential phase were exposed to paraquat or H_2_O_2_ (1 mM and 20 mM) during 1 h (Fig 5). As expected, the higher oxidizing agent concentration led to a more pronounced decrease of the cell viability. Recombinant strains overexpressing the *fldX1* and *fldX2* genes exposed to 1 mM paraquat showed a higher viability (96% and 99%, respectively) than the control strain (68%). After incubation with 20 mM paraquat, the strain overexpressing the *fldX1* gene showed a higher survival (77%) than the control strain (45%). In contrast, the strain overexpressing the *fldX2* gene did not show significant differences in survival in 20mM paraquat (43%) with the control strain. On the other side, during exposure to 1 mM H_2_O_2_, the strain overexpressing the *fldX2* gene (72%) did not show significant differences with the control strain (75%), while the strain overexpressing the *fldX1* gene displayed an unexpected lower viability (52%) than the control strain, suggesting that overexpression of the *fldX1* gene may cause a detrimental effect toward H_2_O_2_ exposure under these conditions. After exposure of the recombinant strains to 20 mM H_2_O_2_, no survival of the recombinant strains was observed (Fig 5).

### Macromolecules damage after exposure to oxidizing agents

**Fig 6:**
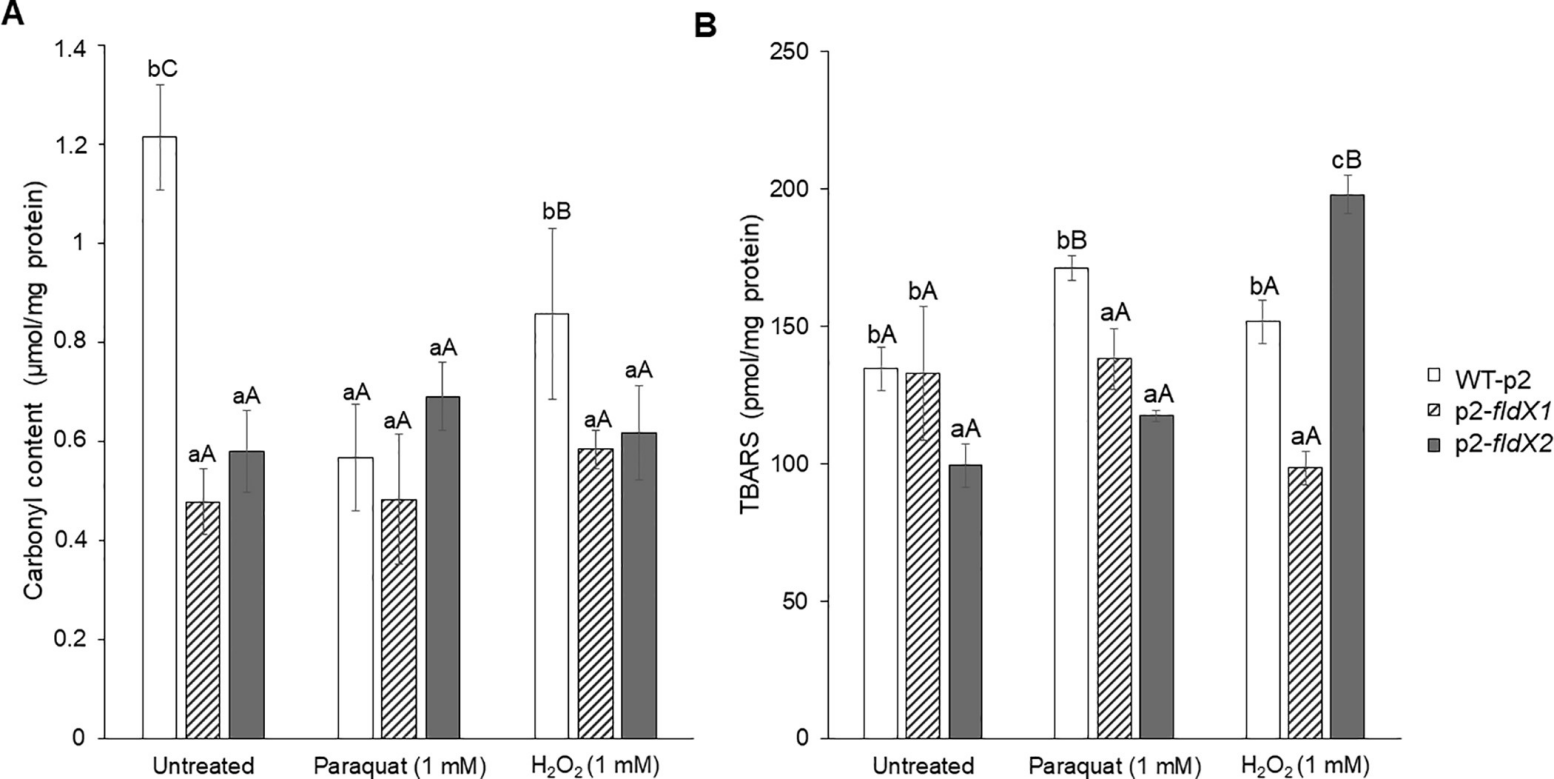
Protective effects of the flavodoxins on macromolecules damage of *P*. *xenovorans* after exposure to oxidizing agents paraquat and H_2_O_2_. (A) For protein carbonylation, nucleic acid-free protein extracts were derivatized with DNPH. After addition of 6 M NaOH, the product was measured spectrophotometrically. (B) Lipid peroxidation was measured by the thiobarbituric acid reactive substances (TBARS) assay. Samples were incubated with TBA for 1 h at 100°C and measured spectrophotometrically. Significant differences were determined with a Tukey Test (p<0.05) as indicated. Lower case letter under the error bars indicate significant differences between strains in each treatment. Capital letter under the error bars indicate significant differences between treatments for each strain. Each value is an average ± SD of at least three independent experiments.

The effects of overexpressing the *fldX1* and *fldX2* genes in *P*. *xenovorans* on damage of macromolecules during exposure to oxidizing agents were determined. Recombinant *P*. *xenovorans* cells grown until exponential phase were exposed to paraquat or H_2_O_2_ (1 mM) during 1 h. As no survival of recombinant cells was observed with H_2_O_2_ 20 mM, only the concentration 1 mM of both oxidizing agents was used in these assays. To quantify carbonyl group content, protein extracts were derivatized with DNPH and the products were measured spectrophotometrically at 450 nm (Fig 6). In absence of the oxidizing agents, *P*. *xenovorans* strains p2-*fldX1* and p2-*fldX2* showed a lower carbonyl content (0.48 μmol/mg of protein and 0.58 μmol/mg of protein, respectively) compared to the control strain (1.21 μmol/mg of protein). For the cells exposed to 1 mM H_2_O_2_, strains p2-*fldX1* and p2-*fldX2* showed less protein carbonylation (0.58 μmol/mg of protein and 0.62 μmol/mg of protein, respectively) than the control strain (0.86 μmol/mg of protein). After paraquat treatment, no significant differences in protein carbonylation were detected between the recombinant strains.

To quantify TBARS, samples reacted with TBA and the products were measured spectrophotometrically at 535 nm (Fig 6). In absence of the oxidizing agents, strain p2-*fldX1* did not showed significant difference with the control strain. However, strain p2-*fldX2* showed less TBARS content (99.43 pmol/mg of protein) compared to the control strain (134.57 pmol/mg of protein). After the exposure to 1 mM paraquat, *P*. *xenovorans* p2-*flX1* (138.20 pmol/mg of protein) and p2-*fldX2* (117.49 pmol/mg of protein) showed lower lipid peroxidation compared to the control strain (171.11 pmol/mg of protein). During exposure of cells to 1 mM H_2_O_2_, strain p2-*fldX1* displayed lower levels of lipid peroxidation (98.49 pmol/mg of protein) than the control strain (151.80 pmol/mg of protein). In contrast, strain p2-*fldX2* showed a higher TBARS content (198.08 pmol/mg of protein) compared to the control strain.

### Gene expression analysis upon exposure to paraquat

**Fig 7:**
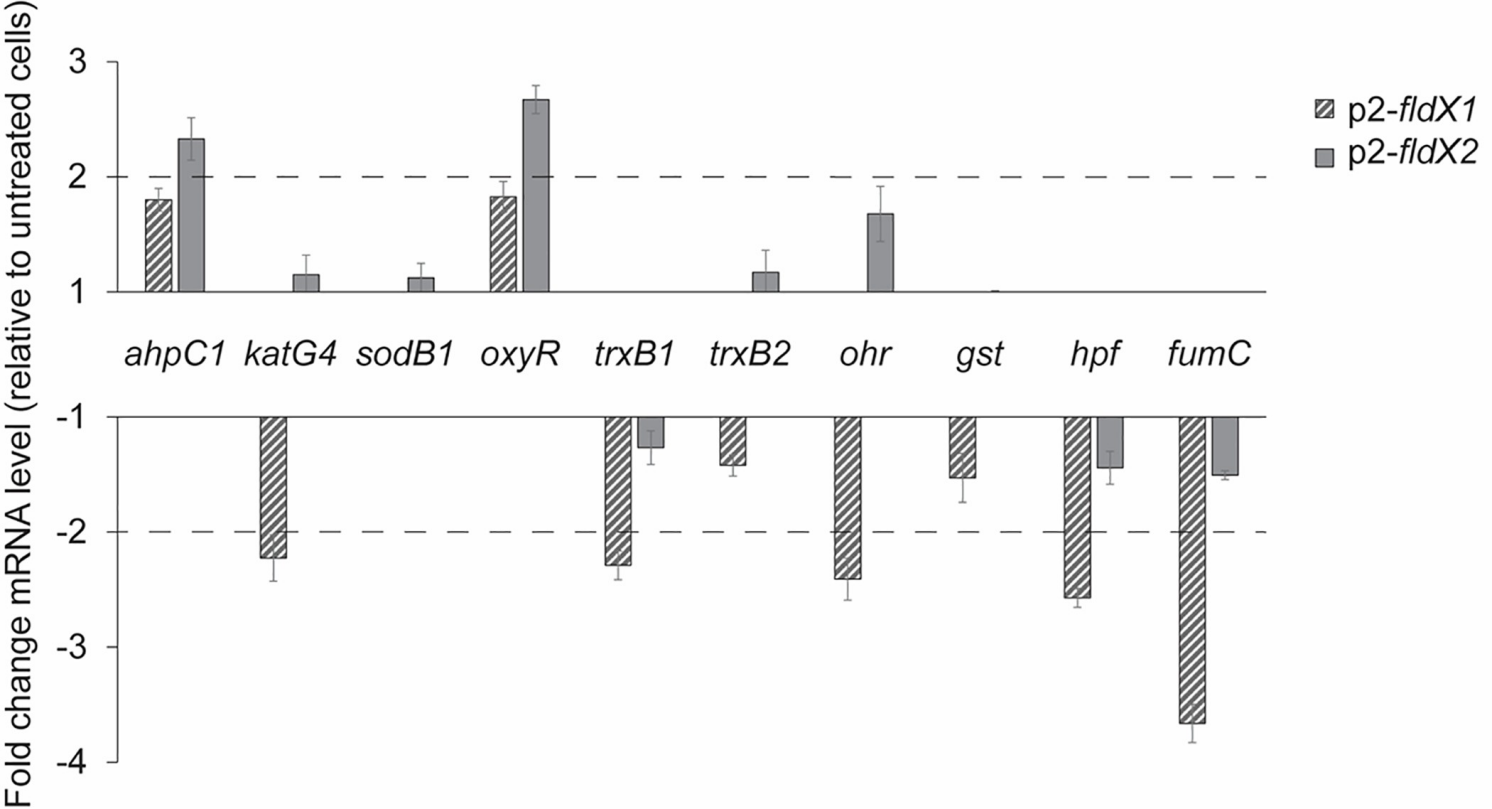
Transcriptional analysis of oxidative stress genes in *P*. *xenovorans* recombinant strains after exposure to paraquat. Cells at exponential phase were exposed to 1 mM paraquat during 1 h. The *ftsZ* and *gyrB* genes were used as reference genes. Significant differences were determined with a Tukey Test (p<0.05) as indicated. Each value is an average ± SD of at least three independent experiments.

The effect of paraquat on the expression of ten specific genes involved in oxidative stress of *P*. *xenovorans* recombinant strains were assessed (Fig 7). These genes were selected based on their high expression in strain LB400 during oxidative stress [35]. Paraquat was selected as the oxidizing agent in these assays, due to higher protection by the overexpression of flavodoxins in *P*. *xenovorans* cells to the exposure of paraquat than H_2_O_2_. A gene expression analysis was carried out by qRT-PCR after exposure of the bacterial cells at exponential phase to 1 mM paraquat. No significant upregulation by paraquat of antioxidant genes was observed in strain *p2-fldX1*. Strain *p2-fldX1* incubated with paraquat presented a downregulation of the *fumC* gene (>3-fold), the *katG4* gene (>2-fold), the *ohr* gene (>2-fold), the *trxB1* gene (>2-fold) and the *hpf* gene (>2-fold) compared to cells incubated in absence of paraquat. The *katG4* gene encodes a catalase, whereas the *fumC* gene encodes a ROS-resistant fumarase. The *ohr* gene encodes an organic hydroperoxide resistance protein, the *trxB1* gene encodes a thioredoxin and the *hpf* encodes a high potential iron-sulfur protein associated to oxidative stress.

Upon exposure to paraquat, recombinant strain p2-*fldX2* showed an upregulation (>2-fold) of the *ahpC1* and *oxyR* genes compared to cells incubated in absence of paraquat. No significant downregulation of antioxidant genes was observed in strain *p2-fldX2*. The *oxyR* gene encodes the H_2_O_2_-sensing transcriptional regulator OxyR. Therefore, the induction of the *oxyR* gene by paraquat suggests increased and threatening intracellular levels of H_2_O_2_ in *p2-fldX2* cells exposed to this oxidizing compound. The *ahpC1* gene encodes the alkyl hydroperoxide reductase involved in detoxification of H_2_O_2_ at low concentration, which is induced in *E*. *coli* by the transcriptional regulator OxyR [45].

In presence of paraquat no changes in expression levels of the *sodB1*, *trxB2* and *gst* genes were observed in both recombinant strains. The *sodB1* gene encodes the scavenging enzyme superoxide dismutase, the *trxB2* gene encodes a thioredoxin, and the *gst* gene encodes a glutathione *S*-transferase.

## Discussion

In this study, two genes encoding flavodoxins (BxeA0278: *fldX1* gene and BxeB0391: *fldX2* gene) were identified in *P*. *xenovorans* LB400 genome and characterized. To study their function, the *fldX* genes were cloned and overexpressed in recombinant strains of *P*. *xenovorans*. The protective effects to oxidative stress induced by superoxide-producing herbicide paraquat and H_2_O_2_ of the expression of these flavodoxins in the recombinant strains p2-*fldX1* and p2-*fldX2* were characterized.

Flavodoxins genes were identified in strain LB400 genome based on the amino acid sequences of previously characterized flavodoxins. The genes *fldX1* (BxeA0278) and *fldX2* (BxeB0391) encoding flavodoxins are located at the major and the minor chromosome, respectively. These flavodoxins share 21.4% identity in amino acid sequence. The FldX1 and FldX2 flavodoxins of strain LB400 possessed two FMN-binding domains (FMN-BD) with similar amino acid length and located at similar positions than flavodoxins of *E*. *coli* DHOl [46], *H*. *pylori* [47] and *Anabaena* sp. PCC 7120 [12]. Long-chain flavodoxins possess a very distinctive secondary structure, consisting of five ß-sheets intercalated with five α-helices, wherein the fifth ß-sheet is divided by a loop of around 20 amino acids [8]. Short-chain flavodoxins possess five ß-sheets intercalated with five α-helices, but without the loop in the fifth ß-sheet, which differentiate them from long-chain flavodoxins. LB400 FldX1 possess the secondary structure of typical long-chain flavodoxins. The absence of the loop at the fifth ß-sheet indicates that FldX2 is a short-chain flavodoxin. Moyano *et al*. [7] reported the secondary structure of the long-chain flavodoxin FldP of *P*. *aeruginosa* PA14, which were compared to FldA of *E*. *coli* and IsiB of *Anabaena* sp. PCC 7119, sharing the same protein domains. The genomic analyses allowed us to identify two flavodoxin-encoding genes in strain LB400 possessing high similarity in length, amino acid sequence, and predicted secondary structure with typical long-chain (FldX1) and short-chain (FldX2) flavodoxins.

In order to study evolutionary distances between bacterial flavodoxins, a phylogenetic analysis was performed. FldX1 and FldX2 of *P*. *xenovorans* LB400 grouped in two different phylogenetic clusters (VII and VI, respectively) within a long-chain flavodoxin cluster (VII) and a short-chain flavodoxin cluster (VI), respectively. The distribution of the short-chain flavodoxin cluster III that includes *Bacillus* flavodoxins, and the cluster V, including the *M*. *elsdenii* and *C*. *beijerinckii* flavodoxins, correlate with the unrooted tree constructed by Pérez-Dorado *et al*. [13]. Previous classification of the short-chain clusters only incorporated Gram-positive bacteria, while the current phylogeny identified the flavodoxins of the Gram-negative *Desulfovibrio desulfuricans* in the same cluster as *M*. *elsdenii* and *C*. *beijerinckii*. The cluster VI contains short-chain flavodoxins homologous to FldX2 and the flavodoxin-4 of *Lactobacillus acidophilus* ATCC 700396. An NMR structure have been performed to the flavodoxin-4 of strain ATCC 700396 identifying a long insertion between α2 and β3 [48], which was also detected in FldX2 amino acid sequence and could be an important feature of this cluster. NifF, IsiB and FldA long-chain flavodoxins were found in the third cluster, recognizing a specific clade for each type as previously described [13]. Although only flavodoxins of the cluster IV have the same biological function among all its members, specific clades include flavodoxins with similar physiological roles (i.e., IsiB and NifF clades). FldX1 belongs to a long-chain flavodoxin cluster VII, closely related to other *Paraburkholderia* and *Burkholderia* orthologs, and to the flavodoxins FldP of *P*. *aeruginosa* PA14 and WrpA of *B*. *abortus*. FldP is the closest flavodoxin with an assigned function to FldX1 (Fig 3). FldP is a 184 amino acid flavodoxin that enhances cell survival during exposure to H_2_O_2_ and the intracellular survival of strain PA14 in mammalian macrophages. Therefore, FldP has been proposed as part of the bacterial adaptation to encounter oxidative stress [7]. The *fldP* gene is present in genomes of some *P*. *aeruginosa* strains, but not all of them, representing an accessory gene for oxidative stress resistance [7]. Interestingly, FldX1, which is phylogenetically related to FldP, showed a higher protection to oxidative damage in *P*. *xenovorans* LB400 than FldX2, suggesting a new clade of flavodoxins within the cluster VII with a role in oxidative stress resistance. It is also interesting to note that flavodoxin FldX2 grouped in the short-chain flavodoxin cluster VI along with the *Lactobacillus acidophilus* flavodoxin-4, whose three-dimensional structure was determined [48].

Aerobic metabolism may produce oxidative stress, mainly by superoxide and H_2_O_2_ accumulation in cells [2]. Superoxide and H_2_O_2_ inactivate metalloenzymes and affect the integrity of DNA and membranes [2]. To investigate the role of LB400 flavodoxins during oxidative stress, the *fldX1* and *fldX2* genes were cloned and overexpressed in *P*. *xenovorans*. Flavodoxin mutants were not generated, as it is well known that the knock-out of genes in *P*. *xenovorans* LB400 is extremely difficult. The *fldX1* gene is expressed >600-fold higher in strain p2-*fldX1* compared to the control strain, whereas the *fldX2* gene is expressed >4000-fold higher in strain p2-*fldX2* than in control strain. Interestingly, the long-chain flavodoxin FldX1 showed a higher protection to oxidative stress in *P*. *xenovorans*, despite their lower expression than the short-chain flavodoxin FldX2.

The long-chain flavodoxin FldX1 of *P*. *xenovorans* showed a high protection towards oxidative stress caused by the superoxide-producing paraquat. Paraquat is a charged quaternary ammonium compound that produces superoxide radical under aerobic growth [40]. It has been reported that exposure to paraquat causes lipid peroxidation in *Streptomyces* sp. M3004 and *E*. *coli* K-12 [49,50]. The redox-cycling compound paraquat generates O_2_^-^ that is dismuted into H_2_O_2_. Then, H_2_O_2_ can reacts with the iron atom of mononuclear iron proteins and iron-sulfur-depending polypeptides, leading to the inactivation of several enzymes involved in Krebs cycle, the pentose phosphate pathway and biosynthetic pathways [51]. Exposure of *E*. *coli sodA* mutant to paraquat decreases the activity of the Krebs cycle iron-sulfur aconitase [52]. After exposure to paraquat (20 mM) on agar plates, strain p2-*fldX1* that overexpress FldX1 showed a significant decrease (~55%) in growth inhibition zones compared to the control strain (Fig 4). Interestingly, strain p2-*fldX1* exhibited also higher survival in presence of paraquat 20 mM (~170%) and 1 mM (~140%) in liquid medium than the control strain (Fig 5). Strain p2-*fldX1* cells displayed a reduction of lipid peroxidation (>20%) compared to control cells after exposure to paraquat (1 mM), but no significant differences in protein carbonylation were detected. In response to paraquat (1 mM), strain p2-*fldX1* downregulated (>2-fold) the *fumC*, *katG4*, *hpf*, *trxB1* and *ohr* genes, but do not significantly upregulated any of the other genes associated to oxidative stress including the key *oxyR* and *ahpC* genes that were studied, suggesting that the oxidative stress response was not activated under these conditions. Overall, these results indicate the strain p2-*fldX1* that overexpress the long-chain flavodoxin FldX1 showed a remarkably resistance to paraquat. Flavodoxins such as FldX1 may replace ferredoxins and scavenge ROS during oxidative stress, but also consume electron donors during paraquat redox cycling [7,8].

In contrast, the overexpression of long-chain flavodoxin FldX1 of *P*. *xenovorans* showed only low protection to oxidative stress induced by the presence of hydrogen peroxide in the medium. A decrease in cell susceptibility on plates and increase in the survival in liquid medium of strain p2-*fldX1* in presence of H_2_O_2_ was not observed. However, strain p2-*fldX1* showed a decrease in protein carbonylation (~30%) and lipid peroxidation (~35%) after exposure to H_2_O_2_ (1 mM). *E*. *coli* strains BW25113 pBAD and KS400 incubated with H_2_O_2_ (0.1 mM) showed 28- and 50-fold higher protein carbonylation, respectively, and similar lipid peroxidation than strain p2-*fldX1* [38,53]. After exposure of *P*. *putida* KT2440 to H_2_O_2_ (1.5 mM), the lipid peroxidation was 4-fold higher than the lipoperoxidation observed in strain p2-*fldX1* [54]. Inhibition of *E*. *coli* growth has been reported to occur at low H_2_O_2_ concentrations (0.03 mM), inactivating key enzymes containing active-site sulfhydryl residues [45]. *P*. *xenovorans* strains, at a higher concentration of H_2_O_2_ (1 mM), exhibited a high survival and lower protein and membrane damage.

The short-chain flavodoxin FldX2 only protected partially to oxidative stress induced by paraquat compared to FldX1, in spite of its higher expression than FldX 1 in recombinant strains. After exposure to paraquat (20 mM) on agar plates, strain p2-*fldX2* do not displayed significant differences in growth inhibition compared to the control strain. Strain p2-*fldX2* showed a higher survival (~150%) than control strain only at the lowest paraquat concentration (1 mM), but not at 20 mM paraquat. Recombinant strain p2-*fldX2* displayed a reduction of lipid peroxidation (~30%) after exposure to paraquat (1 mM), but protein carbonylation did not changed. *P*. *xenovorans* strain p2-*fldX2* showed a significant upregulation by paraquat (1 mM) of the antioxidant *oxyR* and *ahpC1* genes, suggesting increased intracellular levels of ROS (H_2_O_2_) by the superoxide-producing paraquat. H_2_O_2_ activates the expression of the antioxidant *oxyR* and *ahpC1* genes in *P*. *xenovorans* LB400 [35]. On the other side, the overexpression of the short-chain flavodoxin FldX2 showed only slight protection to the exposure of H_2_O_2_. High expression of FldX2 did not influence susceptibility to H_2_O_2_ (20 mM) on agar plates and survival in liquid medium in presence of H_2_O_2_ (1 and 20 mM). Interestingly, strain p2-*fldX2* showed a decrease in protein carbonylation (~30%) after exposure to H_2_O_2_ (1 mM).

Both long-chain flavodoxin FldP, which is closely related to FldX1, and short-chain flavodoxin MioC have been reported in *P*. *aeruginosa* and *P*. *putida* [7]. *P*. *aeruginosa fldP* mutant showed enhanced sensitivity toward H_2_O_2_ toxicity. In contrast, *P*. *aeruginosa mioC* mutant is more tolerant than the wild type strain to oxidative stress induced by paraquat or H_2_O_2_ [55]. Long-chain flavodoxin FldP of *P*. *aeruginosa* enhances survival to oxidizing conditions, such as the exposure to H_2_O_2_ and its intracellular presence in mammalian macrophages [7]. The long-chain flavodoxin FldP is involved in protection against oxidative agents, whereas the role of the short-chain flavodoxin MioC has not yet been elucidated Interestingly, recombinant *E*. *coli*, *P*. *fluorescens* and *Ensifer meliloti* strains overexpressing the long-chain flavodoxin IsiB from *Anabaena* sp. PCC7119 showed a higher survival in presence of paraquat and H_2_O_2_ [3].

Although alkyl hydroperoxide reductase (AhpC) and catalase are responsible for H_2_O_2_ detoxification, it has been demonstrated in *E*. *coli* that Ahp scavenges H_2_O_2_ at low concentrations, while catalase detoxify higher concentrations of hydrogen peroxide [45]. In strains p2-*fldX1* and p2*-fldx2*, no changes were observed in the *katG4* gene expression levels. This may suggest that the intracellular H_2_O_2_ concentration is not high enough to induce the expression of the gene encoding the catalase KatG4. Therefore, hydrogen peroxide may be scavenged by AhpC under these conditions. The *ahpC1* gene is upregulated by paraquat in strain p2*-fldx2*.

Although paraquat is a source of superoxide radical, strains p2-*fldX1* and p2-*fldX2* did not exhibit upregulation of the *sodB1* gene. However, *P*. *xenovorans* LB400 genome possess other three *sodB* homologues (*sodB2*, *sodB3* and *sodB4*), that could be counteracting the superoxide generated by paraquat. The higher viability of strains p2-*fldX1* and p2-*fldX2* in presence of the superoxide-forming paraquat, suggests that FldX1 and FldX2 may be involved in an alternative ROS scavenging-mechanism to thrive oxidative stress. The organic hydroperoxide resistance (Ohr) protein is a thiol-dependent peroxidase induced by organic hydroperoxides [56], such as those generated during lipid peroxidation. Interestingly, TBARS assay indicated a decrease in lipid peroxidation in both *P*. *xenovorans* strains overexpressing flavodoxins, compared to the control strain, which correlates with no upregulation of the *ohr* gene during the incubation with paraquat. The antioxidant *fumC*, *hpf*, *trxB1*, *trxB2* and *gst* genes were not induced in *P*. *xenovorans* recombinant strains exposed to paraquat. Glutathione S-transferases catalyze the conjugation of reduced glutathione to xenobiotic substrates for detoxification [57]. High potential iron-sulfur proteins (HPF) are electron donor that interfere in reactions similar to those of cytochrome C, reestablishing cell redox balance [58]. Thioredoxins participate in disulfide bonds reduction [58]. In *E*. *coli*, the upregulation of the *fumC* gene encoding a class II fumarate hydratase (FumC), occurs during oxidative stress damage, to replace class I iron-sulfur cluster-depending fumarases (FumA and FumB) [2]. Gene expression analysis carried out in hydrocarbon-degrading bacteria revealed the upregulation of antioxidant response genes after exposure to paraquat. *Alcaligenes aquatilis* QD168 displayed an upregulation of two gene copies of *ahpC* (*ahpC1* and *ahpC2*) and two copies of the transcriptional regulator *soxR* (*soxR1* and s*oxR2*) [59]. Exposure of *P*. *putida* KT2440 to paraquat induced expression of a ferredoxin reductase (*fpr*), a superoxide dismutase (*sodA*) and a fumarate hydratase (*fumC-1*) [60].

The *oxyR* and *ahpC* genes were only significantly induced by paraquat in strain p2-*fldX2* and not in strain p2-*fldX1*, indicating that flavodoxin FldX1 exerts a higher protective effect against oxidative stress induced by paraquat. These results also support our hypothesis that FldX1 may be related to an alternative ROS scavenging-mechanism to thrive oxidative stress, which partially involves the OxyR regulon.

Notably, the overexpression of the long-chain flavodoxin FldX1 increased the bacterial growth rate on glucose. In accordance with the improved growth, lower protein carbonylation was observed in strain p2-*fldX1* cells than in control strain cells grown on glucose and not exposed to oxidizing agents (Fig 6). High protein carbonylation was observed in the control strain WT-p2 in absence of oxidizing agents. The selection marker of the plasmids used in this study is kanamycin, which induces hydroxyl radical production, generating an oxidative stress state in *E*. *coli* [61]. Probably kanamycin causes oxidative stress also in *P*. *xenovorans*, which may explain the high protein carbonylation observed. In contrast to FldX1, the overexpression of the short-chain flavodoxin FldX2 did not affect the bacterial growth on glucose. These results suggest an increased fitness of the recombinant strain p2-*fldX1* under these aerobic conditions due to the overexpression of the LB400 long-chain flavodoxin FldX1.

In *E*. *coli*, genes encoding flavodoxins (*fldA* and *fldB*) are induced in the presence of paraquat, as part of the SoxRS regulon, which controls the response to redox-cycling agents [62]. In *P*. *xenovorans*, flavodoxin FldX1 exert a higher effect against paraquat than H_2_O_2_.This may suggest that the gene *fldX1* is regulated by SoxR.

Overall, the overexpression long-chain flavodoxin FldX1 and short-chain flavodoxin FdlX2 exerted a protective role to *P*. *xenovorans* during oxidative stress induced by paraquat and H_2_O_2_, indicating the role of these proteins in the reestablishment of the cell redox balance. Interestingly, FldX1 conferred higher protection than FldX2 to paraquat and H_2_O_2_. This resulted in more resistant cells to paraquat, an increase in viability and a decrease in the macromolecule damage in presence of oxidizing agents.

## Conclusions

In this study, two genes encoding long-chain and short-chain flavodoxins of *P*. *xenovorans* strain LB400 were identified and characterized. A phylogenetic analysis of LB400 flavodoxins indicates that they belong to different evolutionary clusters of long-chain and short-chain flavodoxins. Assays of bacterial susceptibility, survival, macromolecules damage and gene expression indicate that the flavodoxins FldX1 and FdlX2 are involved in the resistance of strain LB400 to oxidative stress caused by paraquat and H_2_O_2_. Several assays indicate that FldX1 and FldX2 are involved in protection against oxidative stress, including (i) the partial protection conferred by FldX1 overexpression to *P*. *xenovorans* susceptibility to 20 mM paraquat on agar plates (Fig 4) (ii) the higher survival by overexpression FldX1 and FldX2 to *P*. *xenovorans* exposed to H_2_O_2_ in liquid medium (Fig 5), (iii) the partial protection conferred by FldX1 and FdlX2 overexpression to *P*. *xenovorans* cells against the protein carbonylation and lipid peroxidation induced by paraquat and H_2_O_2_ (Fig 6) and (iv) the lower upregulation of antioxidant genes in *P*. *xenovorans* cells overexpression FldX1 and FdlX2 in response to paraquat (Fig 7). *P*. *xenovorans* LB400 cells with higher resistance to oxidative stress may be useful to improve the biodegradation of aromatic compounds. Specifically, flavodoxin FldX1 is an attractive protein candidate for subsequent studies to enhance the fitness of *P*. *xenovorans* during the aerobic catabolism of aromatic compounds.
